# An integrated pipeline for high-throughput screening and profiling of spheroids using simple live image analysis of frame to frame variations

**DOI:** 10.1016/j.ymeth.2020.05.017

**Published:** 2021-06

**Authors:** Haneen Alsehli, Fuad Mosis, Christopher Thompson, Eva Hamrud, Erika Wiseman, Eileen Gentleman, Davide Danovi

**Affiliations:** a Centre for Stem Cells & Regenerative Medicine, King’s College London, UK; b National Heart and Lung Institute, Imperial College London, UK; c Micrographia Bio, London, UK; d Centre for Craniofacial and Regenerative Biology, King’s College London, UK; e Stem Cell Hotel, King’s College London, UK

**Keywords:** High content imaging, High throughput imaging, Cell phenotyping, 3D, Spheroids, Stem cells

## Abstract

High-throughput imaging methods can be applied to relevant cell culture models, fostering their use in research and translational applications. Improvements in microscopy, computational capabilities and data analysis have enabled high-throughput, high-content approaches from endpoint 2D microscopy images. Nonetheless, trade-offs in acquisition, computation and storage between content and throughput remain, in particular when cells and cell structures are imaged in 3D. Moreover, live 3D phase contrast microscopy images are not often amenable to analysis because of the high level of background noise.

Cultures of Human induced pluripotent stem cells (hiPSC) offer unprecedented scope to profile and screen conditions affecting cell fate decisions, self-organisation and early embryonic development. However, quantifying changes in the morphology or function of cell structures derived from hiPSCs over time presents significant challenges. Here, we report a novel method based on the analysis of live phase contrast microscopy images of hiPSC spheroids. We compare self-renewing versus differentiating media conditions, which give rise to spheroids with distinct morphologies; round versus branched, respectively. These cell structures are segmented from 2D projections and analysed based on frame-to-frame variations. Importantly, a tailored convolutional neural network is trained and applied to predict culture conditions from time-frame images.

We compare our results with more classic and involved endpoint 3D confocal microscopy and propose that such approaches can complement spheroid-based assays developed for the purpose of screening and profiling. This workflow can be realistically implemented in laboratories using imaging-based high-throughput methods for regenerative medicine and drug discovery.

## Highlights

• Human induced pluripotent stem cells in self-renewing or differentiating conditions.
• Simple live phase images analysed using CellProfiler and frame to frame subtraction.
• Convolutional Neuronal Network predicts culture condition from spheroid morphology.

## Graphical abstract

**Figure:**
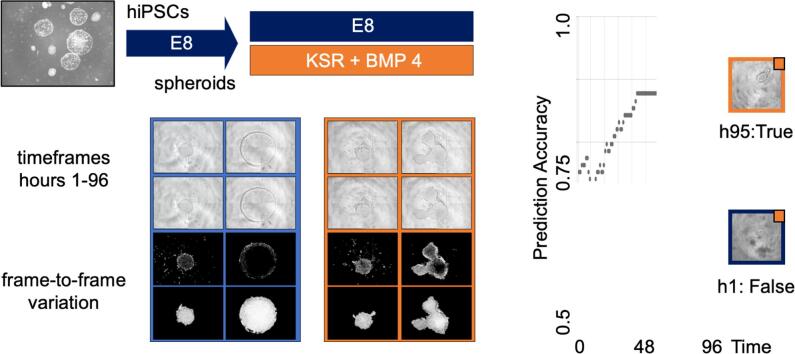

## 1 Description of theoretical basis and framework for the technique

Significant attention has been dedicated to the development of relevant cell culture models that can mirror the behaviour of human cells in vivo. Imaging methods are being deployed as important tools to analyse cells in complex environments in vitro [1]. This has interesting applications in the establishment of quality control protocols for therapeutics, as well as in cell therapy development and manufacturing. In particular, many systems are emerging that enable scientists to observe and quantify cell patterning and the formation of 3D structures, such as spheroids [2]. These applications require the ability to acquire dynamic information over time and ideally perform on-the-fly analyses for quality control, screening and profiling campaigns [3].

High content analysis (HCA) approaches designed to obtain quantitative read-outs from microscopy images provide opportunities to derive automated multi-parametric data to quantify single cell behaviour and morphology. This information can be obtained from both live and endpoint image datasets [4]. There is a clear advantage in combining these two methods especially to study morphogenetic events. Indeed, live imaging yields important time-dependent morphological information despite being more challenging to segment. On the other hand, endpoint images collected from cultures stained with dyes or immunofluorescent cell lineage markers tend to be easier to segment. Quantitative data can be processed via image analysis pipelines and workflows [5], [6]. This approach enables scientists to explore cell dynamics, allowing for insights into biological and biochemical mechanisms in vitro [7], [8].

Continuous improvements in microscopy and computation have effectively empowered high-throughput HCA from endpoint microscopy images in 2D. Nonetheless, trade-offs between content and throughput remain. In particular, quantifying changes in 3D morphology over time is potentially of great interest and yet generally operationally challenging in terms of set up, workflow, data storage and computation. Moreover, live 3D images from phase contrast microscopy tend to prove suboptimal and bring challenges to segmentation. More complex solutions with dyes, reporters and immunofluorescence have been explored and yet are harder to deploy for characterisation of large panels of human cell lines [9]. Furthermore, especially in complex cultures such as hiPSCs and primary cells, studies often focus on either live or endpoint imaging and are rarely combined [5], [7].

hiPSCs have the ability to self-renew (producing identical daughter cells) and to differentiate into virtually all cell types of the human body. These cells offer promising applications for disease modelling and drug discovery. Analysing patterns of cells in vitro has the potential to provide insight into the mechanism of cellular behaviour, cell fate, and early embryonic development [10], [11]. However, significant challenges in acquisition and analysis present when attempting to recapitulate self-organisation, cell fate patterning, and morphogenesis of early mammalian embryogenesis in vitro in 3D and in a dynamic manner [12].

Multicellular aggregates called embryoid bodies (EBs) recapitulate some aspects of in vivo development and facilitate the understanding of cell fate dynamics and organisation [12], [13]. More complex 3D approaches have provided robust simulation of in vivo gastrulation including symmetry breaking-like events prior to differentiation [14], [15]. Methods have been described that dissect the molecular mechanisms involved in gastrulation in manageable in vitro systems that can be referred to as 2.5D [16]. Interesting examples have recently moved the field forward towards predictive modelling via in silico analysis [17]. These methods will have an important value in quality control of cells and could be exploited across a wide range of applications for regenerative medicine [1].

Our eyes effectively combine low resolution frame to frame variation for detection of movements with refined definition and colours. In fact, synergistic strategies have evolved in mammals that combine detection of movements in low lighting conditions (rods for peripheral vision) with higher resolution and colours (cones in the fovea). This combination can be modelled and has in fact been explored for specific purposes in other fields [18]. Similarly in concept, high content analysis strategies have been developed that couple screening lower magnification images with acquisition of a higher magnification images for regions of interest that satisfy defined criteria (see [19], [20]).

Here, we report a method based on frame by frame subtraction, efficiently eliminating areas for which pixel intensities do not vary from frame to frame which in growing spheroids images correspond to the background. This pipeline refines segmentation by considering only the extracted pixels with changes in intensity values from one image to the other in subsequent timeframes. We analyse hiPSC in self-renewing versus differentiating conditions in 96-well plates. We demonstrate that this method can successfully capture distinct morphology variations dependent upon biological conditions in a scalable and high-throughput manner. We demonstrate the value of this approach and propose it can be applied to a range of cell systems presenting similar challenges.

## 2 Materials and methods

### 2.1 Human iPSCs culture

As described [21] 6-well plates were coated with 4% Vitronectin (STEMCELL Technologies) diluted in Phosphate Buffered Saline (PBS, Sigma). Cells were cultured in feeder-free Essential 8 (E8, ThermoFisher) with 2% supplement according to manufacturer’s instructions, and 1% (5000U/ml) Penicillin/Streptomycin (Pen/Strep, Gibco). Cultures were medium-changed daily and passaged every 4 days at approximately 80% confluence. hiPSCs colonies are washed with Hank’s Balanced Salt Solution (HBSS), incubated with Versene cell dissociation solution (Gibco) for 3–4 min at 37 °C, 5% CO_2_ and resuspended in E8 medium in 6-well vitronectin-coated plates. The hiPSC cell line Hoik_1 was obtained from the HipSci biobank (www.hipsci.org) [21].

### 2.2 Preparation of 96 well V-bottom plates

**Fig. 1:**
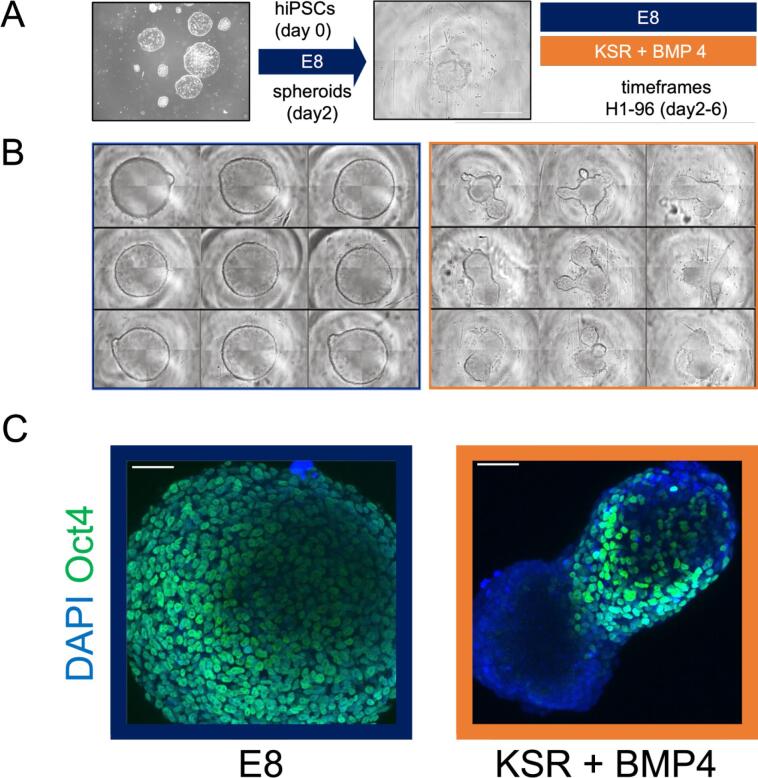
Spheroids obtained from hiPSCs present consistent changes in morphology in different media conditions. (A) Scheme of this study – hiPSC (left) are plated in suspension. After 2 days medium is changed either maintaining in self-renewing E8 conditions or moved to differentiating KSR BMP4 conditions. Wells are imaged every hour from day 2 to day 6. Scale Bar, 500 uM. (B) Consistency of changes in shape – We observe formation of round or branched spheroids in the different media. Representative experiment at 96 h with n = 9 technical replicates. Images included are of spheroids in E8 (left) and KSR BMP4 conditions (right). (C) Endpoint confocal imaging – Representative spheroids at 96 h (endpoint day 6) imaged with confocal microscopy. Note the consistent shape changes in DAPI and staining of Oct4 pluripotency marker.

Before dissociating hiPSCs colonies into single cells and seeding in 96 well V-bottom plates, hiPSCs were observed visually to confirm that they had not undergone spontaneous differentiation as this will affect the spheroid formation and differentiation. To pre-treat the 96 well V-bottom plates, 50 μl of 5% pluronic solution were added before centrifugation for 5 min at 500 × *g*, to ensure the plate is free of bubbles. If bubbles remain we suggest to centrifuge again at higher speed or maximum speed for an additional 5 min. Incubation at RT for 1 h was followed by washing with PBS and addition of 50 μl of E8 medium with 10 μM Y-27632 Rho-kinase inhibitor (ROCKi, ENZO Life Sciences) to each well to avoid drying. This prevents hiPSCs from adhering to the plate and promotes spheroid formation. Note that for the 96 well V-bottom plate layout of this experiment, cells in E8 condition were plated in columns 1–3 whereas cells in KSR-BMP4 condition were plated in columns 10–12. Results in Fig. 1 are from a representative experiment with n = 9 technical replicates. Over 3 biological replicates have been obtained with these conditions in parallel.

### 2.3 Spheroid formation

Cells were washed with HBSS (Gibco), colonies dissociated into single cells by incubating them for 4 min in Accutase (BioLegend) at 37 °C, 5% CO_2_. Single cells were resuspended in E8 and 10 μM ROCKi. Prior to cell seeding, 96 well V-bottom plates (ThermoFisher Scientific) were coated with 5% (w/v) Pluronic solution (Sigma) for 1 h. In these ultra-low adherence conditions, cells were seeded at a density of 750 cells/well in 96 well V-bottom plates, incubated at 37 °C, 5% CO_2_ in E8 and 10 μM ROCKi and left for 24 h to allow cell aggregation. Following another 24 h of culture in E8 medium, hiPSCs cluster and form aggregates. Medium was then replaced with different medium conditions in the presence of 10 μM ROCKi as following: Self-renewing conditions: E8 medium and 1% Pen/Strep; Differentiating conditions: Knock Out Serum Replacement medium (KSR) consisting of Advance DMEM/F-12 medium, supplemented with 20% KnockOut serum replacement, 1% L-Glutamine, 1% Penicillin Streptomycin (5000U/ml) (all Gibco), 0.1 mM β-mercaptoethanol (Sigma), 10 ng/ml basic fibroblast growth factor (bFGF) (Invitrogen) supplemented with 50 ng/ml BMP4 (Invitrogen) morphogen. Culture medium was changed after 48 h once the spheroids formed to the following medium conditions all in the presence of 10 μM ROCKi; E8 medium, and KSR supplemented with (50 ng/ml) BMP4. Subsequently, hiPSCs spheroids were monitored for 96 h using a JuLI™ Stage live imaging microscope in a controlled environment at 37 °C and 5% CO_2_ inside a tissue culture incubator. The plate was spun down for 30 s at 200 × *g* after medium replacement as described in the previous step, to bring all spheroids to the bottom of the plate in the centre of V-bottom wells.

### 2.4 Immunostaining and comparison with end-point analysis

After 96 h, spheroids were fixed using 4% paraformaldehyde (PFA) for 45 min at RT, and washed three times with PBS. Cells were permeabilised with 0.3% Triton X100 in PBS for 1 h at RT, followed by blocking with 5% donkey serum in PBS for 1 h at RT. Primary anti-Oct4 antibody (Abcam) at (1:500) was diluted in 5% donkey serum in PBS and incubated overnight at 4 °C. After three washes with PBS, secondary antibody donkey anti-rabbit Alexa Fluor 488 at (1:500) (Invitrogen), and DAPI at (1:5000) were added and incubated for 1 h at RT in the dark. Spheroid images were acquired using Leica TCS SP8 Confocal laser scanning microscope with a 40x oil objective. Confocal images were analysed in Columbus (Perkin Elmer). Maximum projection for each channel were merged into one image (Calculated Image) smoothed with a Gaussian filter. The resulting image was used to create a mask of the whole organoid (Image Region) and the morphological properties such as area and width to length ratio were measured from these masks. We used proprietary software exclusively to validate the consistency of morphological changes in the spheroids when imaged in more cumbersome endpoint 3D images.

### 2.5 Live imaging

Images of spheroids were obtained by acquiring every hour for 96 h. We tiled 4 fields at 10x objective using the JuLI™ Stage Real-Time Cell History Recorder (NanoEnTek). To image all 96 wells in our conditions takes 18 min. The total time of 96 h (4 days) is calculated for every cycle (1 h). In other words, 96 cycles are acquired in parallel with a shift in time of up to 18 min. Thus, the interval time is calculated for each well and the monitoring of spheroid growth for each well can be considered independent. The difference in time for acquisition of neighbouring fields within the same well is negligible as an entire well is imaged in under 12 s. Selection of the image position is nonetheless critical as it is necessary to ensure that spheroids will be imaged for 96 h. We typically define the central position of the 2 × 2 fields within the well within ample margins accounting for the maximum expected spheroid growth in the following 96 h based on previous experiments. These conditions can be modified for other specific spheroids monitoring needs, other devices and different image acquisitions. Importantly, image acquisition set up (focus, time exposure, and level of brightness) may also vary slightly from one experiment to another and adjustment of focus, brightness level and exposure time are recommended. It is worth noting that this analysis applies to imaging spheroids in transparent material suspension and is more challenging in situations in which this is not the case. Because the image analysis pipelines in this study are based on Delta images as detailed below, we recommend choosing the time-frame intervals that effectively capture growth. In other words, if spheroids growth is not detectable in successive timepoints, longer time intervals should be considered.

### 2.6 Image analysis and segmentation

The image analysis pipeline was created to analyse spheroids’ area and shape using CellProfiler (Broad Institute) software [11]. Initially, raw images are extracted from timepoint 1 to 96, then these images are tiled, batch-loaded and processed using the segmentation pipeline. Here, the differences in pixel intensity values between consecutive images are calculated to identify those that are static. Areas that largely do not change pixel intensities value belong to the background and not the growing spheroid. A subtraction (Delta) is thus performed on every pixel of two consecutive timepoints images. A difference of 0 highlights no change in pixel intensity and therefore no movement between the two time frames, which is classified as the background. Any differences above 1 are classified as a moving spheroid and used to generate the Delta image series. The resultant cell area in this processed Delta image series is segmented to quantify changes in morphology. We then expand the pixels that make up the spheroids as detailed in Appendix 1 (step-by-step description of the CellProfiler pipeline). The rationale is similar to methods described by others [22]. In order to evaluate whether quantitatively, the values of features extracted from our pipeline could be used to cluster spheroids from these two conditions, we performed Principal Component Analysis using Spotfire High Content Profiler (Tibco, PerkinElmer) on individual spheroids imaged over a 96 h time course (see Appendix 2B for the list of morphological features considered).

### 2.7 Spheroid phenotype classification

Images are squared and centred by cropping on the width dimension, using the centroid of the segmented Delta mask as the focal point for each stored image. Data is preprocessed by defining two simple bins based on metadata obtained from the medium conditions: E8 versus KSR+BMP4. The classification model accepts a batch of single-channel grayscale processed images with dimensions of (batch, 1, height, width) and outputs the softmax probability of spheroid type (rounded or branched). The model network is setup as follows. ResNet18 was selected as the backbone of the spheroid classification model, as it has been well-characterised and is available from the PyTorch model module [23]. The ResNet architecture accepts 3-channels RGB images. To accommodate our single channel grayscale images into this architecture, a single 2D convolutional layer was implemented in between the input and ResNet structure. This layer served to expand the input tensor from 1-channel to 3-channels, creating an artificial “RGB” image for input into ResNet. The output layer of ResNet18 was amended to output 2 possible classifications, instead of 1000. Total experiment dataset includes 36 wells, of 96 timepoints each, broken equally among the treatment groups. Datasets for training and evaluation were broken up as follows. A held-out evaluation set was composed of the images from four complete wells from each treatment group (~20% of total samples), selected at random. The training set was composed of the remaining wells from each treatment. For training, standard augmentation was used (random flip, random crop, and resize). Cross-entropy against the binary classification for each image was computed as the loss function, and the ADAM optimiser was used for backpropagation. The model weights were checkpointed regularly and each checkpoint model was evaluated against the held-out evaluation set without augmentation or dropout regularisation. Importantly, the evaluation set was composed of images derived from wells completely excluded from the training set to prevent overfitting via timepoints directly before or after that would have existed in the training set.

## 3 Results

### 3.1 Spheroid formation

Distinct cellular behaviour is observed in the different medium conditions for 3D spheroids (Fig. 1A). In essence, hiPSCs spheroids cultured in KSR+BMP4 medium elongate in shape producing budding and branches. Conversely, cell structures in E8 medium grow to form larger, round spheroids. These structures stain positively for pluripotency marker Oct4 as observed under confocal microscopy (Fig. 1C). The majority of cells are Oct4 positive in round spheroids from E8 conditions whereas only a minority of cells remain pluripotent in KSR+BMP4 conditions and are typically localised in the ‘neck’ of the budding regions. Examples of the shape parameters obtained for these structures from confocal microscopy images are included indicating changes in spheroid morphology parameters (Appendix 2A).

### 3.2 Image analysis

**Fig. 2:**
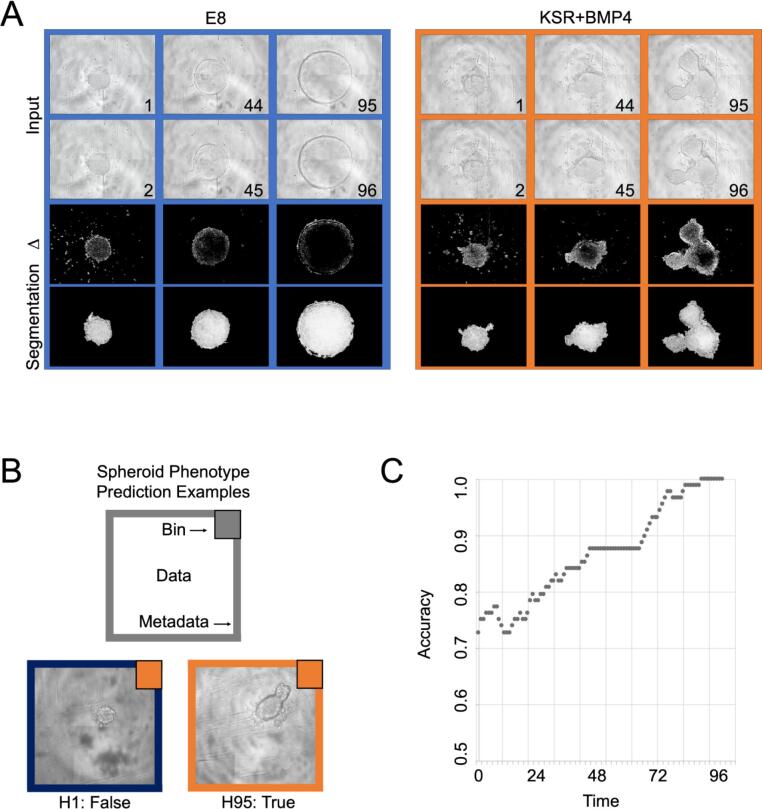
Exploiting live images’ frame-to-frame variations to improve segmentation and automated analysis with computational neural networks. (A) Representative images of spheroids cultured in E8 and KSR+BMP4 media at the beginning (1–2 h), middle (45–46 h), and end (95–96 h) of the observation period. The image Delta is produced by subtraction of pixel intensities: note that background halos surrounding the spheroids are effectively removed with this strategy. Segmentation is obtained from the Delta images via a dedicated CellProfiler pipeline (see Appendix 1 for details). Scale Bar, 500 um. (B) colours refer to prediction (top right square) and actual condition (frame); one early timepoint example is inaccurately classified, whereas one late timepoint example is correct. (C) A tailored Convolutional Neural Network is trained and used to predict Spheroid Phenotype Classification in two classes based on metadata of the medium conditions used. The graph shows phenotype prediction accuracy (rolling average over 10 h) over time increasing at later timepoints.

Having consistently observed such morphologies in structure from these diverse conditions in endpoints, we set to evaluate whether simple live imaging could be used instead of confocal endpoint analysis. To quantify the phenotypic features variations over time, including size and shape of hiPSCs we developed a dedicated image analysis workflow within the framework of the open source CellProfiler software [11]. An image series, termed Delta, was generated by quantifying the differences in pixel intensity values of consecutive images within a time frame. This strategy efficiently subtracts the background from one image to the other (Fig. 2A). Morphological features from the segmented regions, such as area and form factor, were captured for each timepoint and Principal Component Analysis of all features is shown over time (Appendix 2B). This indicates that as time progresses from 1 h to 96 h the spheroids diverge presenting specific morphological parameters. Altogether these observations prompted us to explore whether the information retained with simple microscopy over time would be sufficient to predict using an automated approach the conditions of culture of the specific spheroid. We used the Delta segmentation images to guide cropped box-shaped image datasets and trained a convolutional neural network by presenting images assigned to two bins of round/E8 versus branched/KSR+BMP4 cell structures. Examples of predicted erroneous and correct classifications are given (Fig. 2B, insets). Confusion is present in early timepoints which appear to be almost random. Conversely, the binning gradually becomes more accurate as the morphology of the spheroids in the diverse medium conditions becomes more distinct over time (Fig. 2C).

## 4 Conclusion

We propose a novel method to exploit frame to frame variation for efficient segmentation of simple phase contrast microscopy for live 3D spheroids. This increases significantly the speed and hence the throughput compared to existing strategies based on analysis of endpoints. A CellProfiler based pipeline is coupled with a trained convolutional network to predict distinct media conditions analysing morphology. This self-contained method is validated by unsupervised clustering using principal component analysis and by comparison with 3D confocal microscopy. In this study, spheroids are obtained from hiPSCs. A broad range of application across diverse cell systems in regenerative medicine and drug discovery can be pursued. We recommend such approaches can be immediately adapted and efficiently implemented by laboratories using imaging-based high-throughput methods.

## CRediT authorship contribution statement

**Haneen Alsehli:** Conceptualization, Data curation, Formal analysis, Funding acquisition, Investigation, Methodology, Project administration, Validation, Visualization, Writing - original draft. **Fuad Mosis:** Conceptualization, Funding acquisition, Methodology, Software. **Christopher Thompson:** Software. **Eva Hamrud:** Data curation, Visualization. **Erika Wiseman:** Data curation, Formal analysis, Visualization. **Eileen Gentleman:** Supervision. **Davide Danovi:** Conceptualization, Funding acquisition, Methodology, Project administration, Resources, Supervision, Writing - original draft, Writing - review & editing.

## Figures and Tables

**Table:** 

Module	Function	Purpose	Output
i. Primary analysis			
Image math	Calculates absolute difference in pixel intensity between each frame and the following frame to generate a new set of images.	Filters out all pixels defined as background, with no change in intensity from each frame to the following frame in the stack.	
Save images	Exports images as a new stack (Delta) for further analysis.	Exports a new set of images as a stack (Delta) that can be analysed.	
Identify primary objects	Filters out objects to only retain those between 1 and 5000 pixels via thresholding.	Identifies objects (areas of pixels intensities that make up the spheroid).	
Expand or shrink objects	Expands each object by 2 pixels.	Allows all objects including the ones that make up the spheroid to be in contact with each other.	
Split or merge objects	Merges all objects that are in contact with each other (i.e. have a distance in pixels of 0).	Transforms all small objects that make the spheroid into a single object.	
Fill objects	Holes smaller than an area of 800 pixels will be filled.	Fills up left-over holes within the object (spheroid).	
Measure object size shape	Identifies the size of all objects.	Identifies the size of all remaining objects in the field, including the spheroid.	
Filter objects	Removes all objects with an area of less than 60,000 pixels.	Filters out anything not large enough to be a spheroid.	
Expand or shrink objects	Shrinks object by 2 pixels.	Returns the edge of the spheroid to its original size after initial 2-pixel expansion.	
ii. Extract features			
Measure object size shape	Measures the morphology properties of the object.	Measures features of object (spheroid), e.g. area, form factor.	
Export to spreadsheet	Exports measurements to a spreadsheet for analysis.	Allows subsequent analysis of exported values for desired features.	
iii. Extract segmented spheroid stack			
Convert objects to image	Converts object to an image, generating a stack of binary spheroid (termed Binary).	Generates an image from the object (currently a binary form) to extract spheroid from Delta stack.	
Image math	Calculates absolute difference in pixel intensity between each frame of Delta against Binary, which generates a set of images of background frames (termed Background).	Uses Binary spheroid image stack to remove spheroid from Delta, thus creating a stack containing only the Background from Delta.	
Image math	Calculates absolute difference in pixel intensity between each frame of Delta and Background to generate a set of images (termed Segmented).	Removes background of spheroid from Delta, and generates a stack of images with a completely segmented spheroid.	
Save images	Exports Segmented as a stack.	Exports Segmented stack to be subsequently analysed.	

**Figure:**
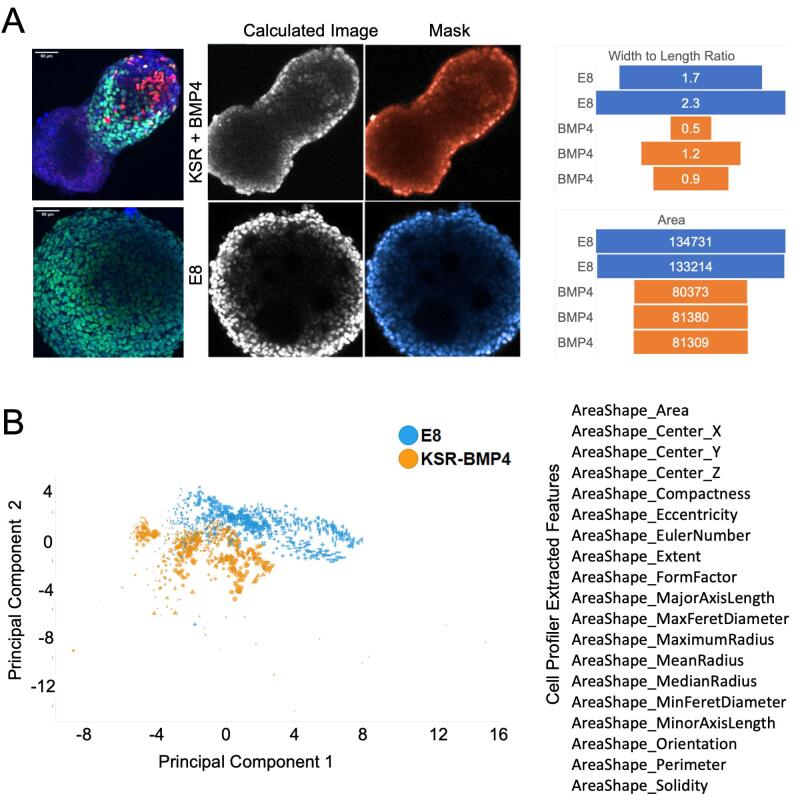


## Appendix 1 Detailed CellProfiler pipeline

Step-by-step description of the CellProfiler pipeline modules to facilitate application of the described strategy to specific readers needs.CellProfiler pipeline and example dataset included in Supplementary.

## Appendix 2 Validation with proprietary software

Validation of morphology change in endpoint (Columbus) (A) For the examples images in Fig. 1B, Maximum projection DAPI staining is segmented with Columbus as Mask from Calculated Image. Morphological parameters Width to Length Ratio (top) and Area (bottom) are shown for representative spheroids analysed to highlight and quantify morphological differences between conditions. (B) Validation of morphology features value change (Spotfire High Content Profiler, Tibco, PerkinElmer). Multidimensional reduction on morphology features extracted from live image analysis through the CellProfiler pipeline allows separation of spheroids based on the different conditions. Principal Component Analysis for segmented images at each timepoint for E8 (blue) or KSR+BMP4 (orange). Size of the data points represents time; shape represents different wells. Unsupervised clustering based on morphology parameters becomes apparent after several hours.
