# Supplementary figures and images for: An integrated pipeline for high-throughput screening and profiling of spheroids using simple live image analysis of frame to frame variations
Source: Methods. 2021 Jun;190:33–43. doi: 10.1016/j.ymeth.2020.05.017 (PMC8165939; doi:10.1016/j.ymeth.2020.05.017)

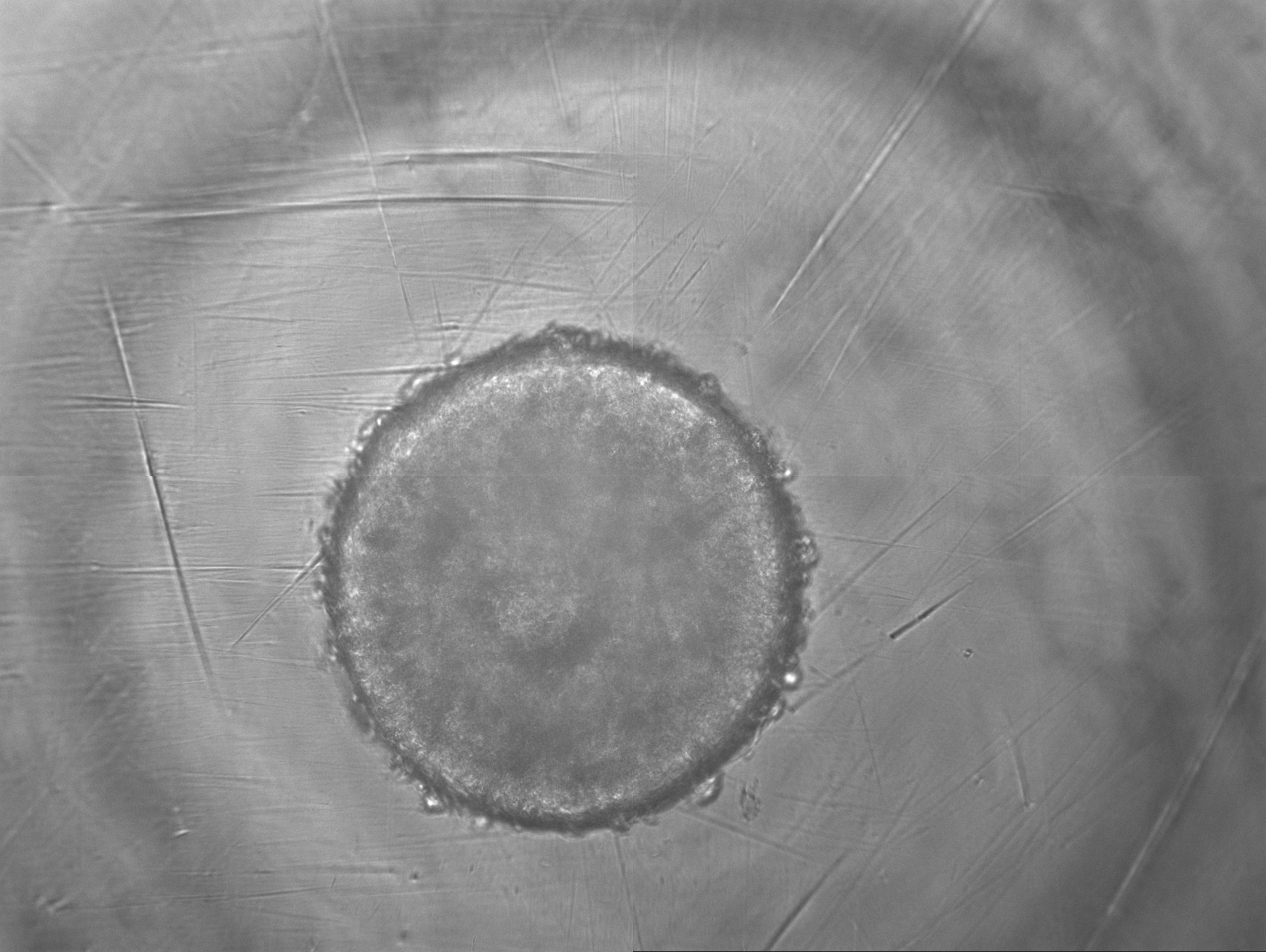

Supplement: Supplementary Data 2 [file mmc2.zip › E8-Spheroid95.tiff]

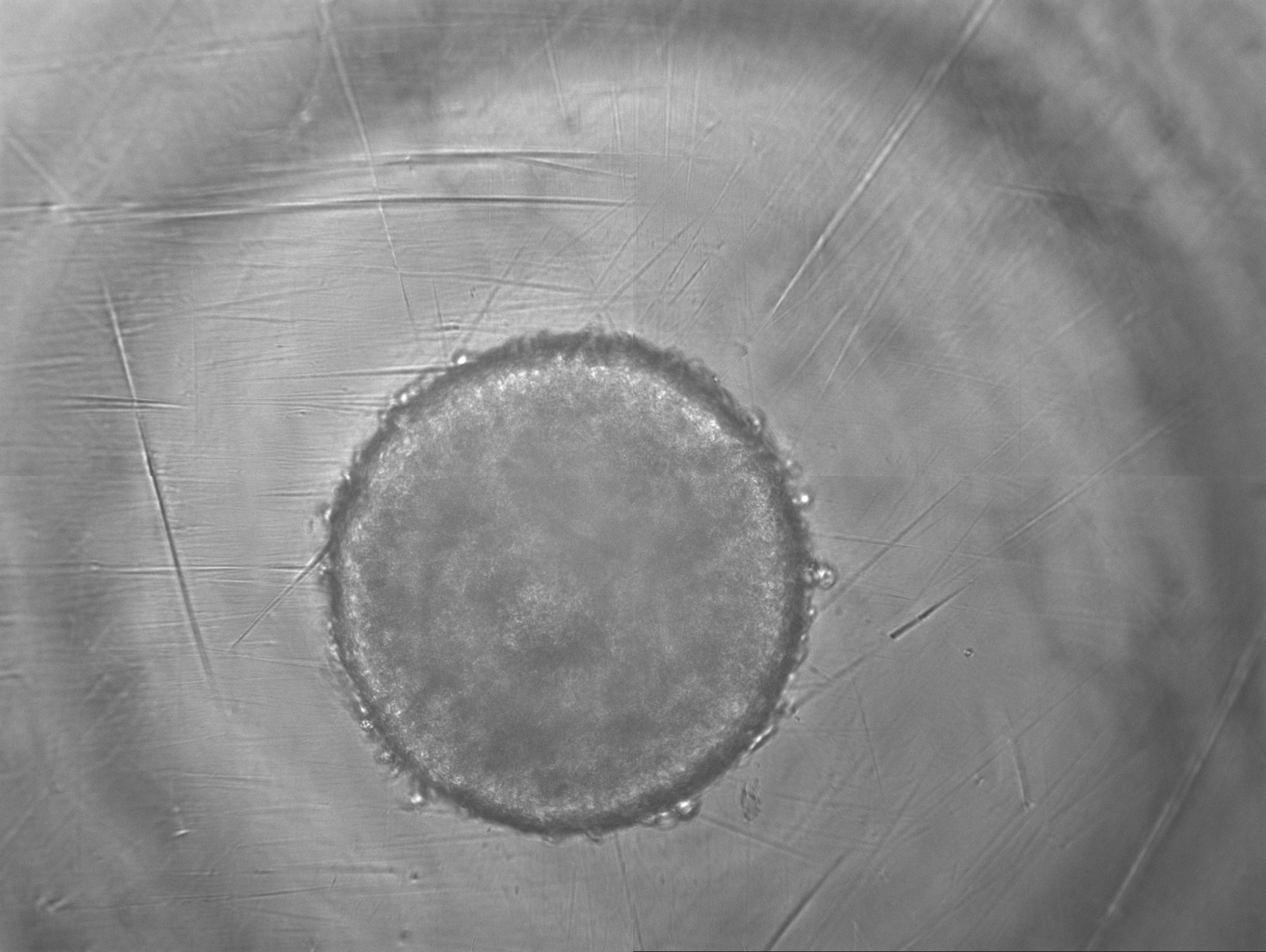

Supplement: Supplementary Data 2 [file mmc2.zip › E8-Spheroid96.tiff]

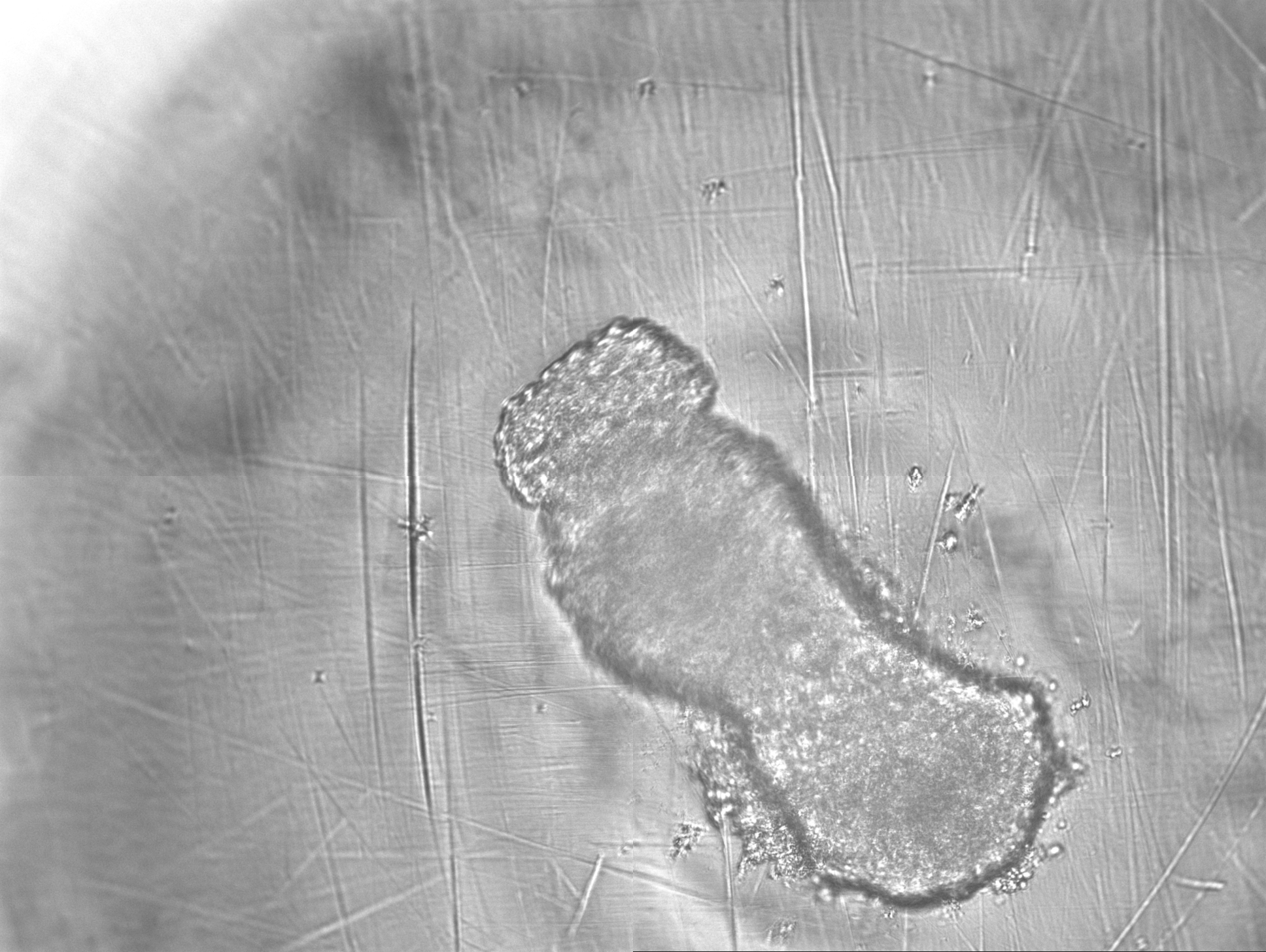

Supplement: Supplementary Data 2 [file mmc2.zip › KSR-BMP4-Spheroid95.tiff]

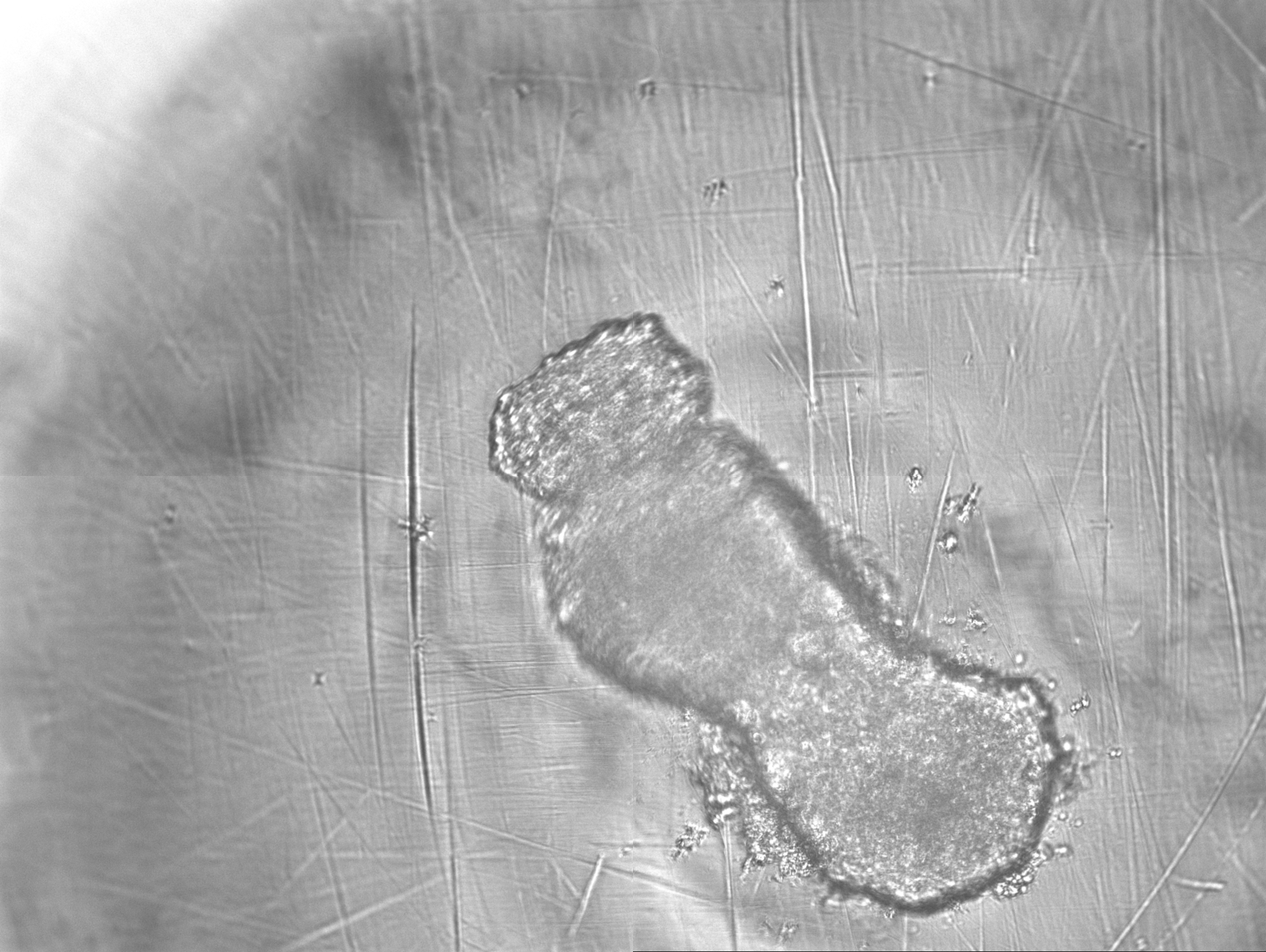

Supplement: Supplementary Data 2 [file mmc2.zip › KSR-BMP4-Spheroid96.tiff]
